# Effect of Shortening the Scan Duration on Quantitative Accuracy of [^18^F]Flortaucipir Studies

**DOI:** 10.1007/s11307-021-01581-5

**Published:** 2021-01-26

**Authors:** Hayel Tuncel, Denise Visser, Maqsood Yaqub, Tessa Timmers, Emma E. Wolters, Rik Ossenkoppele, Wiesje M. van der Flier, Bart N. M. van Berckel, Ronald Boellaard, Sandeep S. V. Golla

**Affiliations:** 1 Department of Radiology & Nuclear Medicine, Amsterdam Neuroscience, Vrije Universiteit Amsterdam, Amsterdam UMC, De Boelelaan 1117, 1081 HV Amsterdam, The Netherlands; 2 Alzheimer Center Amsterdam, Department of Neurology, Amsterdam Neuroscience, Vrije Universiteit Amsterdam, Amsterdam UMC, Amsterdam, The Netherlands; 3 Clinical Memory Research Unit, Lund University, Lund, Sweden; 4 Department of Epidemiology and Data Sciences, Vrije Universiteit Amsterdam, Amsterdam UMC, Amsterdam, The Netherlands

**Keywords:** [^18^F]Flortaucipir, PET, Alzheimer’s disease

## Abstract

**Purpose:**

Dynamic positron emission tomography (PET) protocols allow for accurate quantification of [^18^F]flortaucipir-specific binding. However, dynamic acquisitions can be challenging given the long required scan duration of 130 min. The current study assessed the effect of shorter scan protocols for [^18^F]flortaucipir on its quantitative accuracy.

**Procedures:**

Two study cohorts with Alzheimer’s disease (AD) patients and healthy controls (HC) were included. All subjects underwent a 130-min dynamic [^18^F]flortaucipir PET scan consisting of two parts (0–60/80–130 min) post-injection. Arterial sampling was acquired during scanning of the first cohort only. For the second cohort, a second PET scan was acquired within 1–4 weeks of the first PET scan to assess test-retest repeatability (TRT). Three alternative time intervals were explored for the second part of the scan: 80–120, 80–110 and 80–100 min. Furthermore, the first part of the scan was also varied: 0–50, 0–40 and 0–30 min time intervals were assessed. The gap in the reference TACs was interpolated using four different interpolation methods: population-based input function 2T4k_V_B_ (POP-IP_2T4k_V_B_), cubic, linear and exponential. Regional binding potential (BP_ND_) and relative tracer delivery (R_1_) values estimated using simplified reference tissue model (SRTM) and/or receptor parametric mapping (RPM). The different scan protocols were compared to the respective values estimated using the original scan acquisition. In addition, TRT of the RPM BP_ND_ and R_1_ values estimated using the optimal shortest scan duration was also assessed.

**Results:**

RPM BP_ND_ and R_1_ obtained using 0–30/80–100 min scan and POP-IP_2T4k_V_B_ reference region interpolation had an excellent correlation with the respective parametric values estimated using the original scan duration (*r*^2^ > 0.95). The TRT of RPM BP_ND_ and R_1_ using the shortest scan duration was − 1 ± 5 % and − 1 ± 6 % respectively.

**Conclusions:**

This study demonstrated that [^18^F]flortaucipir PET scan can be acquired with sufficient quantitative accuracy using only 50 min of dual-time-window scanning time.

**Supplementary Information:**

The online version contains supplementary material available at 10.1007/s11307-021-01581-5.

## Introduction

Dynamic positron emission tomography (PET) scan protocols allow for accurate quantitative measures [1, 2] of specific binding of PET tracers. Moreover, dynamic scan protocols yield additional information about functional measures such as perfusion [3]. Semi-quantitative measures from static scans are usually sufficient for clinical application, but accurate quantification of tracer uptake is of major importance in the context of early-stage pathology, clinical trials [1] and longitudinal studies. Some PET tracers like the tau tracer [^18^F]flortaucipir require a long acquisition period because of the slow tracer kinetics. This can be challenging, especially when working with a vulnerable population (like patients with Alzheimer’s disease (AD)).

*In vivo* quantification of tau pathology is important because intracellular accumulation of hyperphosphorylated tau proteins into neurofibrillary tangles (NFTs) is one of the pathological hallmarks of AD [4]. Indeed, histopathological studies have shown that the amount of NFTs correlate well with the severity of their cognitive symptoms during life [5, 6]. [^18^F]Flortaucipir is worldwide the most widely used PET tracer for detecting and quantifying these NFTs. For the analysis of [^18^F]flortaucipir scans, most studies prefer semi-quantitative measures due to their practical applicability and computational simplicity [7–9]. However, studies involving dynamic imaging provided more accurate and precise pharmacokinetic parameters and provide estimates for relative tracer delivery (R_1_) or relative cerebral blood flow (rCBF) [2, 10–15], which is important for monitoring flow changes. For instance, a study by van Berckel et al. [16] observed that longitudinal changes in [^11^C]PIB standardized uptake value ratio (SUVr) do not reflect changes in specific [^11^C]PIB binding but rather are secondary to changes in blood flow during the natural course of AD.

Our group has performed dynamic acquisition of [^18^F]flortaucipir scans, using a 130-min dual-time-window dynamic scan protocol including a 20-min break (after the first 60 min of acquisition) [17–21]. Several aspects are of importance to obtain a reliable protocol with reduced overall scanning time. Firstly, the scan must include the wash-in of the tracer and tissue peak activity to be able to assess the tracer influx into the tissue. In addition, tracer efflux information is also necessary to be able to estimate the tracer efflux back to plasma and the specific binding compartment. The second part ideally has to contain the 80–100 min interval to calculate SUVr, since this is the internationally conventional SUVr interval for [^18^F]flortaucipir [22]. So, the new scanning protocol needs to include an early part of the tracer kinetics and also at least 80–100 min post-injection (p.i.), implying that a dual-time-window protocol should be used. Scanning time can be shortened by increasing the gap of the dual-time-window. Interpolation is needed to fill this gap in the time activity curve (TAC) of the reference region to be able to perform reference tissue model–based tracer kinetic modelling. Therefore, the aim of the study is to investigate whether a shorter overall scan duration for [^18^F]flortaucipir PET dual-time-window scans is feasible, while retaining quantitative accuracy.

## Methods

### Study Sample

For the current project, two study cohorts were included. The first cohort consisted of ten biomarker (PET/CSF)-confirmed AD patients and ten cognitively normal controls who underwent a 130-min dynamic [^18^F]flortaucipir PET scan with arterial sampling (“full kinetic model cohort”). Subject characteristics have been described previously [18]. The second cohort consisted of eight subjects with AD and six cognitively normal controls that underwent two 130-min dynamic [^18^F]flortaucipir PET scans within a time interval of minimum 1 week, and maximum 4 weeks (“test-retest cohort”). The subject characteristics have been described previously [19]. The current study was approved by the Medical Ethics Committee of the Amsterdam University Medical Center. All subjects signed an informed consent form prior to participation.

### Scan Procedures

T1-weighted MRI scans were acquired for all participants using a 3.0 T Philips Ingenuity Time-of-Flight PET/MR scanner (Philips medical systems, Best, the Netherlands). Isotropic structural 3D T1-weighted images were obtained using a sagittal turbo field echo sequence (1.00 mm^3^ isotropic voxels, repetition time = 7.9 ms, echo time = 4.5 ms, flip angle = 8°) for brain tissue segmentation.

All subjects from the full kinetic model cohort underwent a 130-min dynamic [^18^F]flortaucipir PET scan on a Gemini TF-64 PET/CT scanner (Philips Medical Systems, Best, The Netherlands) with continuous arterial sampling after administration of 223 ± 18 MBq of [^18^F]flortaucipir. Details described elsewhere [17–19]. Subjects from the test-retest cohort underwent two 130-min dynamic [^18^F]flortaucipir PET scans on a Philips Ingenuity TF PET/CT scanner after administration of [^18^F]flortaucipir (237 ± 15 MBq at test and 245 ± 18 MBq at retest) as described in detail previously [19]. In short, a low-dose CT for attenuation correction was acquired, followed by a 60-min dynamic (brain) emission scan initiated simultaneously with tracer injection. After a 20-min break, a second low-dose CT was acquired before an additional dynamic emission scan during the interval 80–130 min p.i. During scanning, the head of the subjects was stabilized to reduce movement artefacts. Furthermore, subjects were positioned within the centre of axial and transaxial fields of view, such that the orbitomeatal line was parallel to the detectors with the use of laser beams.

For the full kinetic model cohort, continuous arterial blood sampling, using an online detection, [23] was collected during 60-min p.i. PET acquisition. Furthermore, manual arterial samples were collected at set time points (5, 10, 15, 20, 40, 60, 80, 105 and 130 min p.i.) to measure plasma metabolite fractions and plasma-to-whole-blood ratios. Using the aforementioned information, the continuous online blood sampler data was calibrated and corrected for metabolites, plasma-to-whole-blood ratios and delay, providing a metabolite-corrected arterial plasma input function. In addition, whole-blood input function was obtained for blood volume correction.

### Image Processing

PET scans were reconstructed with a matrix size of 128 × 128 × 90 and a final voxel size of 2 × 2 × 2 mm^3^. All standard corrections were applied. During processing of the PET scans, first part and second part of the scan were checked for motion, separately. Thereafter, both the PET scan sessions were co-registered into a single dataset of 29 frames (1 × 15, 3 × 5, 3 × 10, 4 × 60, 2 × 150, 2 × 300, 4 × 600 and 10 × 300 s) using Vinci software (Max Plank Institute, Cologne, Germany). The last 10 frames belonged to the second PET session.

Structural 3D T1-weighted MRI images were co-registered to the PET images also using Vinci software (Max Plank Institute, Cologne, Germany). The Hammers template [24], which is incorporated in PVElab [25], was used to delineate regions of interest (ROIs) on the co-registered MR scan and superimposed onto the dynamic PET scan to obtain regional time activity curves (TACs). All 68 cortical and subcortical regions from the Hammer template were included. Regional TACs extracted from the PET scans were analysed using a reversible 2-tissue compartment model with blood volume correction (2T4k_V_B_) and simplified reference tissue model (SRTM) [26]. Receptor parametric mapping (RPM) [27] and standardized uptake value ratios (SUVr) were used to obtain parametric images. Cerebellum grey matter (obtained from PVElab) was used as the reference region.

### Shortening the Second Part of the Scan (80–130 Min P.I.)

In these analyses, the first part of the scan remained 0–60 min p.i. The second part of the scan was shortened; three shorter time intervals were explored: 80–120 min, 80–110 min and 80–100 min. For each subject, shortened PET scans were acquired by removing 2 to 6 frames to reach the specified scan intervals. Reference region TACs were extracted from these shortened PET scans to estimate kinetic parameters. BP_ND_ and R_1_ values were estimated using RPM from the three different scan durations (0–60/80–100, 0–60/80–110 and 0–60/80–120 min). RPM-derived regional BP_ND_ and R_1_ values were compared to the corresponding non-linear regression (NLR)-based SRTM-derived BP_ND_ and R_1_, and plasma input–derived distribution volume ratio (DVR) values from the original scan duration (0–60/80–130 min). The optimal shortened time interval for the second part was used and fixed during subsequent evaluation of scan shortening of the first part of the PET scan.

### Shortening the First Part of the Scan (0–60 Min P.I.)

For shortening the first part of the scan, three time intervals were explored: 0–50, 0–40 and 0–30 min p.i, all in combination with 80–100 min scan interval for the second part of the imaging protocol. For each subject, the corresponding frames were removed to obtain the PET scans with these specified time intervals.

The original scan duration had a gap of only 20 min; the gap in the reference region was interpolated by using cubic interpolation. The larger gap (> 20 min) in the new dual-time-window protocol results in more missing data points in the reference TAC for which proper interpolation is required. Therefore, four different interpolation methods were assessed: population-based plasma input function in combination with a reversible two-tissue compartmental model with blood volume correction (POP-IP_2T4k_V_B_) to fit the reference tissue TAC, standard cubic interpolation, linear interpolation, and interpolation based on fitting an exponential function to the TAC (excluding points until peak uptake). All scripts were built in house using MATLAB (version R2017B, MathWorks, USA).

The POP-IP_2T4K_V_B_ interpolation method was based on using the population-averaged metabolite-corrected plasma input function and a reversible two-tissue compartmental model with blood volume correction (2T4k_V_B_). A 2T4k_V_B_ model was used, since it was evaluated in the previous studies [28] that this model best describes the *in vivo* kinetics of [^18^F]flortaucipir. So based on the previous research, it was assumed that the cerebellum presents a 2T4k_V_B_ kinetics and the cerebellum TAC with the gap was fitted using this model and the population-averaged metabolite-corrected plasma input function. The fit was visually examined for certainty and the gap in the cerebellum TAC was filled using the values from the fitted curve.

SRTM-derived BP_ND_ and R_1_ estimates using the shortened scan durations and the four different interpolated reference region TACs were obtained. These regional parametric values were compared to the corresponding NLR-based reference region and plasma input–derived values obtained using the original scan duration (0–60/80–130 min).

BP_ND_ and R_1_ parametric images were acquired for the optimally shortened scans with interpolated reference region (using the optimal interpolation technique(s)). Regional parametric values were extracted from these parametric images and were compared to corresponding values derived using plasma input–based and reference tissue–based NLR and RPM from the original scan duration (0–60/80–130 min).

SUVr using the interval 80–100 min (SUVr_(80−100 min)_) was also evaluated. Regional SUVr values obtained from this time interval were compared with the respective quantitative parameters (DVR, SRTM BP_ND_ and RPM BP_ND_) estimated using the original scan duration (0–60/80–130 min).

### Test-Retest Repeatability Analysis

For the test-retest repeatability (TRT) analysis, the test-retest cohort was used. The TRT of RPM BP_ND_ and R_1_ values derived from the optimal shortened scan duration were compared to the test-retest repeatability of RPM BP_ND_ obtained using the original scan duration (0–60/80–130 min). In addition, TRT for SUVr_(80–100)_ was also assessed. The TRT was calculated using Eq. 1.

1 $$ \mathrm{TRT}\ \left(\%\right)=\frac{\left(\mathrm{Retest}\ \mathrm{value}-\mathrm{Test}\ \mathrm{value}\right)\ }{\left(\mathrm{Retest}\ \mathrm{value}+\mathrm{Test}\ \mathrm{value}\right)} \times 200 $$

### Statistical Analysis

Linear regression fitting and correlation coefficients (*r*^2^) were used to compare BP_ND_ and R_1_ for the shortened scan durations and SUVr_(80−100 min)_ against corresponding parametric values for the original scan duration (0–60/80–130 min) derived from plasma input–based and reference tissue–based NLR and RPM. Furthermore, Bland-Altman plots were used to assess and illustrate TRT performance.

## Results

### Shortening the Second Part of the Scan (80–130 Min P.I.)

The RPM BP_ND_ values obtained from the three shortened scan durations (0–60/80–120, 0–60/80–110 and 0–60/80–100 min) provided excellent correlations with plasma input DVR-1, SRTM BP_ND_ and RPM BP_ND_ obtained using the original acquisition time window (0–60/80–130) (Table 1; all *r*^2^ > 0.93). Reduction of the time interval of the second part to 100 min had negligible effects on the RPM BP_ND_ estimation: correspondence to DVR-1 (HC: *r*^2^ = 0.94, slope = 0.95; AD: *r*^2^ = 0.94, slope = 0.92), SRTM BP_ND_ (HC: *r*^2^ = 0.98, slope = 1.05; AD: *r*^2^ = 0.96, slope = 0.85) and RPM BP_ND_ (HC: *r*^2^ = 0.98, slope = 1.04; AD: *r*^2^ = 0.99, slope = 0.94). Comparison of regional SRTM BP_ND_ values obtained using the shorter time interval (0–60/80–100) to plasma input DVR-1 and SRTM BP_ND_ obtained with the original scan duration (0–60/80–130) are presented in Supplementary Table 1.

**Table 1.** RPM BP_ND_ obtained using shorter time intervals compared to plasma input DVR-1, SRTM BP_ND_ and RPM BP_ND_ obtained with the original scan duration

	DVR-1 (0–60/80–130)				SRTM BP_ND_ (0–60/80–130)				RPM BP_ND_ (0–60/80–130)			
	HC		AD		HC		AD		HC		AD	
	*r*^2^	Slope	*r*^2^	Slope	*r*^2^	Slope	*r*^2^	Slope	*r*^2^	Slope	*r*^2^	Slope
RPM BP_ND_ (0–60/80–120)	0.95	0.91	0.95	0.96	0.99	1.02	0.97	0.90	1.00	1.01	1.00	0.99
RPM BP_ND_ (0–60/80–110)	0.95	0.92	0.95	0.94	0.99	1.03	0.97	0.88	0.99	1.02	1.00	0.97
RPM BP_ND_ (0–60/80–100)	0.94	0.95	0.94	0.92	0.98	1.05	0.96	0.85	0.98	1.04	0.99	0.94

The correlation and slope for the original scan duration between RPM BP_ND_ and DVR-1 was *r*^2^ = 0.95 slope = 0.91 for HC and, *r*^2^ = 0.96 slope = 0.98 for AD. The correspondence between original RPM BP_ND_ and SRTM BP_ND_ was *r*^2^ = 1.00 slope = 1.01 for HC and *r*^2^ = 0.97 slope = 0.91 for AD

The RPM R_1_ values obtained from the three shortened scan durations (0–60/80–120, 0–60/80–110 and 0–60/80–100) also provided excellent correlations with SRTM R_1_ and RPM R_1_ estimated using the original acquisition time window (0–60/80–130) (Supplementary Table 2).

### Shortening the First Part of the Scan (0–60 Min P.I.)

**Fig. 1.**
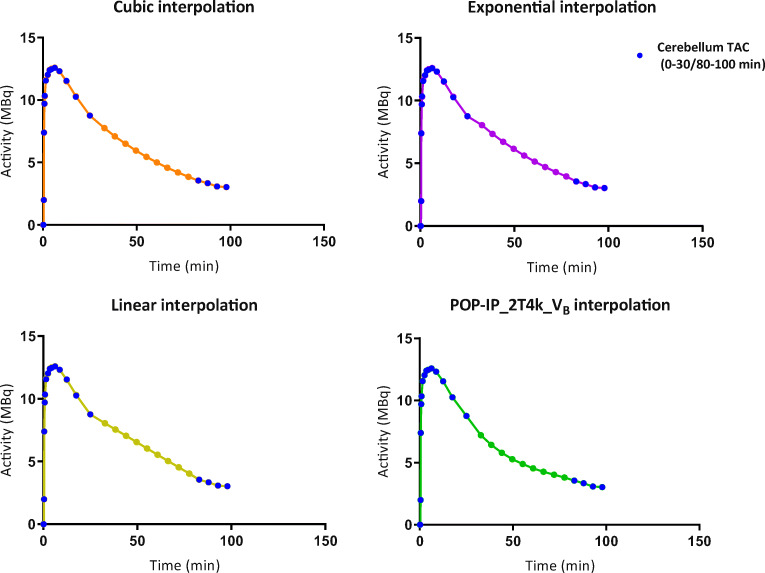
Interpolation of the gap in reference region TAC (30 to 80 min) with different interpolation methods.

In Fig. 1, the different interpolations of a typical reference TAC for the shortest dual-time-window (0–30/80–100 min) assessed in this study are presented. For all shortened scan durations, SRTM BP_ND_ using the reference TACs interpolated with either POP-IP_2T4k_V_B_ or cubic interpolation methods had the best correspondence with plasma input DVR-1 and SRTM BP_ND_ (*r*^2^ > 0.90, Table 2) obtained with the original scan duration. Reduction of the time interval of the first part of the scan to 30 min and using POP-IP_2T4k_V_B_ for reference region interpolation had negligible effects on the quantitative accuracy of the estimated kinetic parameters with respect to that estimated using the original scan duration: DVR-1 (HC: *r*^2^ = 0.93, slope = 0.94; AD: *r*^2^ = 0.92, slope = 0.97) and SRTM BP_ND_ (HC: *r*^2^ = 0.96, slope = 1.02; AD: *r*^2^ = 0.98, slope = 0.89). SRTM BP_ND_ values obtained with 0–30/80–100 min data using cubic interpolation for reference region had similar agreement with DVR-1 (HC: *r*^2^ = 0.94, slope = 0.91; AD: *r*^2^ = 0.91, slope = 0.92) and SRTM BP_ND_ (HC: *r*^2^ = 0.96, slope = 0.98; AD: *r*^2^ = 0.98, slope = 0.85) from the original scan duration. Good correlations were observed for linear and exponential interpolation methods (*r*^2^ > 0.90, Table 2). However, these interpolation methods resulted in higher underestimation (15–25 %) of the parametric values.

**Table 2.** Shortened time intervals interpolated using four different methods are compared with plasma input DVR-1 and SRTM BP_ND_ obtained with the original scan duration

		DVR-1 (0–60/80–130)				SRTM BP_ND_ (0–60/80–130)			
		HC		AD		HC		AD	
		*r*^2^	Slope	*r*^2^	Slope	*r*^2^	Slope	*r*^2^	Slope
POP-IP 2T4k_V_B_	SRTM BP_ND_ (0–50/80–100)	0.96	0.96	0.91	1.01	0.98	1.04	0.99	0.93
	SRTM BP_ND_ (0–40/80–100)	0.95	0.96	0.91	0.99	0.98	1.03	0.99	0.91
	SRTM BP_ND_ (0–30/80–100)	0.93	0.94	0.92	0.97	0.96	1.02	0.98	0.89
Cubic	SRTM BP_ND_ (0–50/80–100)	0.96	0.94	0.91	1.01	0.99	1.02	0.99	0.93
	SRTM BP_ND_ (0–40/80–100)	0.96	0.95	0.91	0.98	0.99	1.02	0.99	0.90
	SRTM BP_ND_ (0–30/80–100)	0.94	0.91	0.91	0.92	0.97	0.98	0.98	0.85
Linear	SRTM BP_ND_ (0–50/80–100)	0.96	0.93	0.91	0.98	0.99	1.02	0.99	0.90
	SRTM BP_ND_ (0–40/80–100)	0.96	0.95	0.91	0.92	0.98	1.03	0.99	0.84
	SRTM BP_ND_ (0–30/80–100)	0.94	0.91	0.91	0.84	0.96	0.98	0.98	0.76
Exponential	SRTM BP_ND_ (0–50/80–100)	0.96	0.94	0.91	1.01	0.99	1.02	0.99	0.93
	SRTM BP_ND_ (0–40/80–100)	0.96	0.96	0.90	0.97	0.98	1.04	0.98	0.90
	SRTM BP_ND_ (0–30/80–100)	0.94	0.92	0.90	0.92	0.96	0.99	0.97	0.85

The correspondence of SRTM BP_ND_ with DVR-1 for the original scan duration was *r*^2^ = 0.96, slope = 0.90 for HC, and *r*^2^ = 0.93, slope = 1.09 for AD subjects

**Fig. 2.**
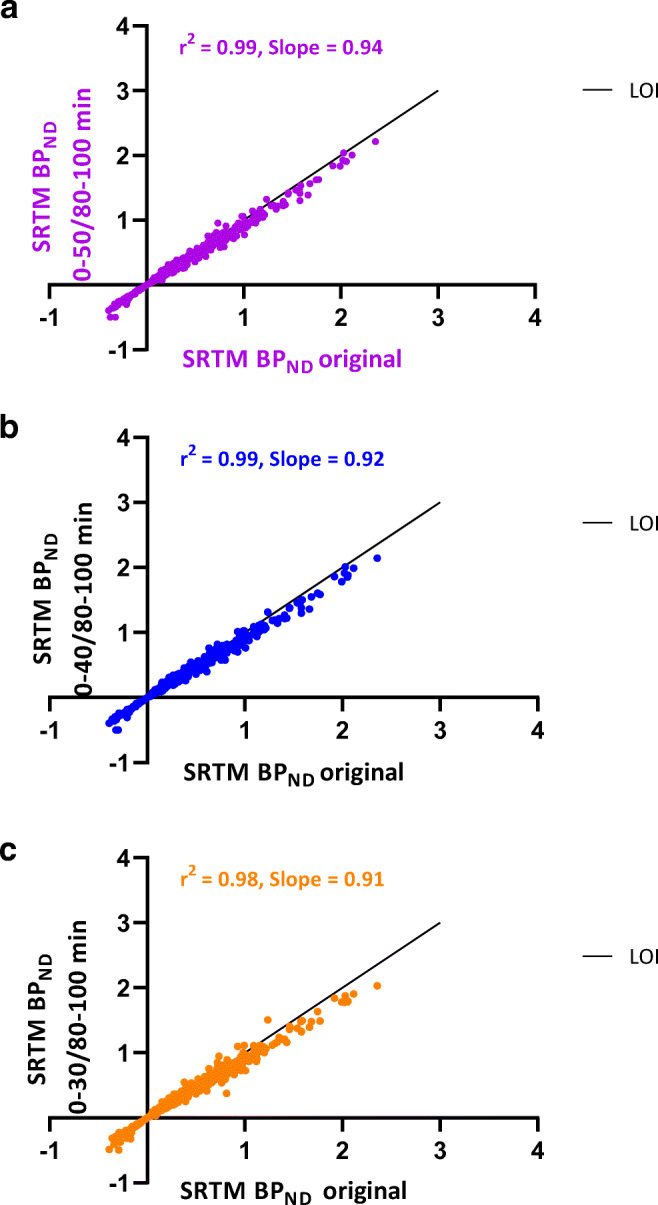
Comparison of SRTM BP_ND_ estimated using shortened time intervals **a** 0–50/80–100 min, **b** 0–40/80–100 min, **c** 0–30/80–100 min and POP-IP_2T4k_V_B_ interpolation for reference region against SRTM BP_ND_ obtained from the original scan duration (0–60/80–130 min). LOI, line of identity.

Figure 2 presents the correspondence of SRTM BP_ND_ values obtained with the shortened scan durations using POP-IP_2T4k_V_B_ for reference region interpolation against SRTM BP_ND_ values estimated from the original scan duration. The bias increased as the first part was shortened. An underestimation of 9 % was observed for SRTM BP_ND_ values with the shortened scan duration for both groups (0–30/80–100 min) with respect to that obtained with original scan duration. SRTM R_1_ values derived from the shortened scan duration (0–30/80–100 min) showed excellent correlations with SRTM and RPM R_1_ values obtained with the original scan duration for HC and AD patients for each interpolation method (*r*^2^ > 0.95, Supplementary Table 3).

**Fig. 3.**
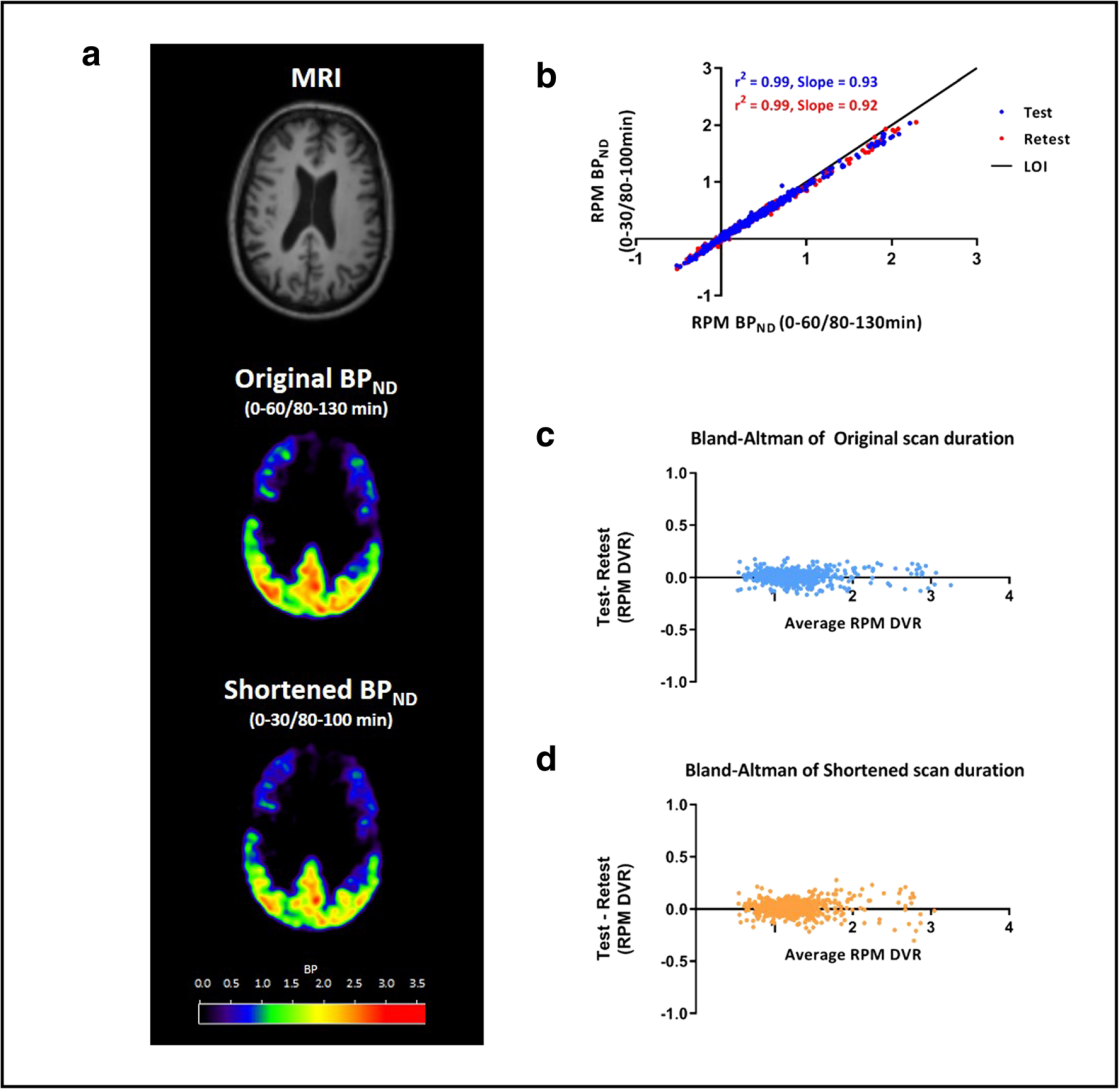
**a** An example of the BP_ND_ images for a representative AD patient are displayed for the original scan (0–60/80–130 min) duration and shortened scan duration (0–30/80–100 min using POP-IP_2T4k_V_B_ interpolation) along with the corresponding MR. **b** Comparison of BP_ND_ obtained from the shortened scan duration (0–30/80–100 min using POP-IP_2T4k_V_B_ interpolation) against BP_ND_ obtained with the original scan duration (0–60/80–130 min). **c** Bland-Altman plot of the original test-retest differences for RPM DVR (BP_ND_+1) values. **d** Bland-Altman plot of the test-retest differences for RPM DVR (BP_ND_+1) values using shortened scan duration (0–30/80–100 min) and POP-IP_2T4k_V_B_ method for reference region interpolation.

An example of the RPM BP_ND_ images for the original scan duration and shortened scan duration (0–30/80–100) is illustrated in Fig. 3a. Comparison of RPM BP_ND_ obtained from the shortest scan duration (0–30/80–100 min) using POP-IP_2T4k_V_B_ for reference region interpolation against RPM BP_ND_ obtained with the original scan duration is shown in Fig. 3b and Supplementary Fig. 1. RPM BP_ND_ obtained with the shortest scan duration (0–30/80–100 min) using either POP-IP_2T4k_V_B_ or cubic methods for reference region interpolation and SUVr_80-100 min_ compared to original DVR-1, SRTM BP_ND_ and RPM BP_ND_ values are shown in Table 3 for HC and AD patients separately. The same comparisons were made for RPM R_1_ and are illustrated in Supplementary Table 4.

**Table 3.** Comparison of RPM BP_ND_ obtained with the shortest scanning interval (0–30/80–100) and SUVr_(80-100 min)_ to plasma input DVR-1, SRTM BP_ND_ and RPM BP_ND_ derived from the original scan duration (0–60/80–130)

		DVR-1 (0–60/80–130)				SRTM BP_ND_ (0–60/80–130)				RPM BP_ND_ (0–60/80–130)			
		HC		AD		HC		AD		HC		AD	
		*r*^2^	Slope	*r*^2^	Slope	*r*^2^	Slope	*r*^2^	Slope	*r*^2^	Slope	*r*^2^	Slope
POP-IP 2T4k_V_B_	RPM BP_ND_ (0–30/80–100)	0.94	0.94	0.95	0.93	0.98	1.04	0.97	0.84	0.98	1.03	0.99	0.95
Cubic	RPM BP_ND_ (0–30/80–100)	0.94	0.93	0.94	0.90	0.98	1.03	0.97	0.81	0.98	1.02	0.99	0.92
	SUVr (80–100)	0.80	0.95	0.92	1.10	0.90	1.15	0.93	0.98	0.87	1.11	0.96	1.13

The correlation and slope for the original scan duration between RPM BP_ND_ and DVR-1 was *r*^2^ = 0.95 slope = 0.91 for HC, and *r*^2^ = 0.96 slope = 0.98 for AD. The correspondence between original RPM BP_ND_ and SRTM BP_ND_ was *r*^2^ = 1.00 slope = 1.01 for HC, and *r*^2^ = 0.98 slope = 0.88 for AD

### Test-Retest Repeatability

The RPM BP_ND_ values estimated for both test and retest scans using the shortest assessed scan duration (0–30/80–100) and POP-IP_2T4k_V_B_ or cubic interpolation for the reference region correlated well (Table 4). The test-retest parametric correlations using the short scan duration and interpolated reference TAC (POP-IP_2T4k_V_B_ and cubic interpolation methods) were similar to the correlations using the original scan duration. The TRT across all Hammers’ regions of interest for RPM BP_ND_ was − 1 % ± 4 for HC and 0 % ± 4 for AD patients using the original dual-time-window acquisition (Table 4). Furthermore, TRT for R_1_ was 0 % ± 6 for both groups using the original dual-time-window acquisition. The TRT for BP_ND_ remained the same for HC when using the shortest dual-time-window 0–30/80–100 min with either of the interpolations methods (POP-IP_2T4k_V_B_ or cubic interpolation). For AD patients, the TRT changed to 0 % ± 5 when using the shortest dual-time-window 0–30/80–100 min with POP-IP_2T4k_V_B_ or cubic interpolation. The TRT for R_1_ remained similar as with the original data (0 % ± 6) for the shortest dual-time-window (0–30/80–100 min) both with POP-IP_2T4k_V_B_ and cubic interpolation for both groups. TRT for SUVr_80-100 min_ was − 1 % ± 5 for HC and − 1 % ± 6 for AD patients (Table 4).

**Table 4.** RPM BP_ND_, RPM R_1_ and SUVr values obtained from the test scan are compared to corresponding values obtained from the retest scan for the original scan duration (0–60/80–130), and for the shortened scan duration (0–30/80–100) interpolated with cubic or POP-IP_2T4k_V_B_ interpolation method

	HC			AD		
	*r*^2^	Slope	%TRT	*r*^2^	Slope	%TRT
RPM BP_ND_ (0–60/80–130)	0.91	0.95	− 1 ± 4	0.98	1.0	0 ± 4
Cubic RPM BP_ND_ (0–30/80–100)	0.90	0.96	− 1 ± 4	0.98	0.98	0 ± 5
POP-IP_2T4k_V_B_ RPM BP_ND_ (0–30/80–100)	0.90	0.96	− 1 ± 4	0.97	1.0	0 ± 5
RPM R_1_ (0–60/80–130)	0.86	0.95	0 ± 6	0.94	0.96	0 ± 6
Cubic RPM R_1_ (0–30/80–100)	0.86	0.94	0 ± 6	0.94	0.96	0 ± 6
POP-IP_2T4k_V_B_ RPM R_1_ (0–30/80–100)	0.86	0.95	0 ± 6	0.94	0.96	0 ± 6
SUVr (80–100)	0.86	0.97	− 1 ± 5	0.96	1.05	− 1 ± 7

Bland-Altman plots for the RPM BP_ND_+1 illustrating TRT for all Hammers’ regions obtained with the original scan duration and the shortest scan duration with POP-IP_2T4k_V_B_ reference region interpolation are presented in Fig. 3c and d.

## Discussion

The current study demonstrated that for [^18^F]flortaucipir expanding the break in the dual-time-window protocol with just a 50-min overall scanning time (early interval of 0–30 min, than a coffee break, followed by a late interval of 80–100 min) had minimal effect on the quantitative accuracy. The optimal shortened dual-time-window protocol (0–30/80–100 min) allows sufficiently accurate estimation of BP_ND_ while reducing patient burden and enables interleaved scanning, where other patients could use the camera during breaks within the scan period.

An excellent correlation was observed between the shortened acquisition protocol (0–30/80–100 min) and the original dual-time-window (0–60/80–130 min) protocol. Four different interpolation methods were used to interpolate the missing data between the two time windows for the reference region TAC (cerebellum grey matter). According to our results, POP-IP_2T4k_V_B_ interpolation, which uses a population-averaged plasma input function, showed a good correspondence of the estimated kinetic parameters to that obtained from the original scan protocol, and the lowest under/over-estimation(s) compared to other interpolation methods. Heeman et al. [29] showed that POP-IP_2T4k_V_B_ interpolation method also works well to interpolate the missing data points in a dual-time-window protocol for [^18^F]flutemetamol and [^18^F]florbetaben. They concluded that the introduction of a gap with a maximum of 60 min in a dual-time-window protocol (early interval of 0–30 min followed by a late interval of 90–110 min) does not affect quantitative accuracy for [^18^F]flutemetamol and [^18^F]florbetaben. As such, POP-IP_2T4k_V_B_ interpolation does not only work well for [^18^F]flortaucipir but also for [^18^F]flutemetamol and [^18^F]florbetaben, possibly because the model describes the *in vivo* kinetics of the tracers best and is therefore ideal for estimating the missing reference region data points accurately.

The correlations for all interpolation methods were comparable (Table 2). This was not expected, since linear and exponential interpolation did not follow the course of tracer as can be observed in Fig. 1. A possible explanation could be that the gap between the dual-time-windows was not substantially large enough to see significant differences in correlations between the interpolation methods. However, a substantial underestimation (at times up to 20 % or even more) was observed in AD patients for the shortened SRTM BP_ND_ values obtained with linear and exponential interpolation methods when compared to plasma input DVR-1 and SRTM BP_ND_ obtained with the original scan duration. This indicates that these interpolation methods are not suitable for quantitatively accurate kinetic parameter estimation for [^18^F]flortaucipir. For SRTM BP_ND_ values obtained with POP-IP_2T4k_V_B_ interpolation, the biases remained within ~ 10 % for the same comparisons (Table 2). Comparisons of RPM BP_ND_ obtained with the shortest scanning interval (0–30/80–100) to plasma input DVR-1, SRTM BP_ND_ and RPM BP_ND_ derived from the original scan duration (0–60/80–130) showed that POP-IP_2T4k_V_B_ interpolation had a minimal and acceptable bias of ~ 5 % (Table 3). Since RPM BP_ND_ is the main parameter outcome for [^18^F]flortaucipir, shortening the scan duration to 0–30/80–100 min with POP-IP_2T4k_V_B_ interpolation for the reference region will provide quantitative acceptable accurate results. However, when individual regions are assessed, relatively higher bias (~ 8 %) was observed in regions with higher tau uptake (RPM DVR > 2) (Supplementary Fig. 1).

From Table 3, it is evident that SUVr_(80-100 min)_ presents over-/underestimations when compared to DVR-1, SRTM BP_ND_ and RPM BP_ND_ (using 0–60/80–130 min scan duration data). In contrast, parameters estimated using a shortened scan duration data had a much better correspondence with the parameters estimated using the whole scan duration data (0–60/80–130 min). Although with a shortened scan duration, a 50-min scanning time is required, which is 30 min more than that required for a static scan; SUVr is still semi-quantitative. Moreover, it is known that SUVr might be effected by blood flow changes overtime, and hence is an unreliable parameter for longitudinal studies, whereas using the shortened dual-time-window protocol (0–30/80–100), a flow estimate (R_1_) to evaluate the effect of flow can be estimated and therefore is apt for longitudinal studies. Moreover with the proposed method, only 50 min of actual scanning time is required on contrast to 110 min of actual scan duration (decreasing the patient burden by 60 min of scanning).

Dynamic [^18^F]flortaucipir PET scans can be used to estimate both the specific binding of the tracer to tau pathology (BP_ND_) and as well as rCBF (R_1_) using RPM. Perfusion imaging is a reliable biomarker to assess neuronal dysfunction in neurodegenerative diseases [30]. R_1_ is an estimate of the relative blood flow, and it has been shown that it has a high correlation with [^18^F]FDG uptake [30] and so an accurate estimation of R_1_ is also necessary. The current study demonstrated that shortening the scan duration to 0–30/80–100 min had negligible effects on R_1_ estimations. As such, it can be safely assumed that the scanning protocol can be reduced to 0–30/80–100 min with minimal bias (~ 7 % on average). If the implementation for POP-IP_2T4k_V_B_ interpolation is not possible, use of cubic interpolation is a reliable alternative to interpolate the gap between the dual-time-windows for the reference region.

Unfortunately, further reduction of the acquisition time for the first part was not possible since the bias in the estimated parameters was increasing as the scan duration was being reduced (Fig. 2). A reason could be that as the peak approaches, further loss of information makes it difficult to estimate the efflux kinetics of [^18^F]flortaucipir. Further reduction of the second part of the scan was also not possible, since we want to be able to compare with the internationally mostly used 80–100-min SUVr time interval.

The shortened dual-time-window (0–30/80–100 min) acquisition protocol provided almost identical TRT values when compared to the observed TRT values for the original scan protocol (0–60/80–130) (Fig. 3). This implies that the shortened dual-time-window protocol not only provides quantitatively acceptable estimates but also result in high repeatability, suggesting that it can be reliably used for longitudinal and treatment-monitoring studies. However, the benefits of using a dynamic scanning protocol over static scanning protocol in a longitudinal setup for [^18^F]Flortaucipir need further validation, even though it has been presented by van Berckel et al. [16] that SUVr does get affected by changes in flow and under-/overestimates the underlying specific binding in case of [^11^C]PIB. Making use of a dual-time-window protocol also has some challenges to consider. Even if the total acquisition time is reduced with 60 min, it still takes longer than a single static acquisition protocol used for SUVr. Another disadvantage of using a dual-time-window protocol is the added CT attenuation scan for the second part of the scan, which is still present in this new shortened scanning protocol. Furthermore, the pre-processing of the PET images are more demanding compared to simplified methods, for instance, due to a required additional co-registration of the first part to the second part of the scan. Yet, the main advantage is that, apart from obtaining quantitative information on BP_ND_, this protocol also generates parametric R_1_ data that may be used as a surrogate for flow and/or FDG uptake [15] and this protocol could therefore obviate the need to make a separate FDG scan, when clinically required.

## Conclusion

The current study demonstrated that quantitatively acceptable [^18^F]flortaucipir kinetic parameters can be obtained using just 50 min of total scanning time by implementing a dual-time-window protocol (0–30/80–100 min). The best method to interpolate the gap in the reference tissue is 2T4k_V_B_ tracer kinetic model with population-averaged metabolite-corrected plasma input function. Reducing the dual-time-window protocol enables interleaved scanning, reduces patient burden and enables efficient use of tracer batches and cost-effectiveness.

## Supplementary Information

ESM 1 (PNG 162 kb)

High Resolution Image (EPS 1912 kb)

ESM 2 (DOCX 12 kb)

ESM 3 (DOCX 12 kb)

ESM 4 (DOCX 13 kb)

ESM 5 (DOCX 12 kb)
