# Supplementary figures and images for: Effect of Shortening the Scan Duration on Quantitative Accuracy of [18F]Flortaucipir Studies
Source: Mol Imaging Biol. 2021 Jan 26;23(4):604–13. doi: 10.1007/s11307-021-01581-5 (PMC8277654; doi:10.1007/s11307-021-01581-5)

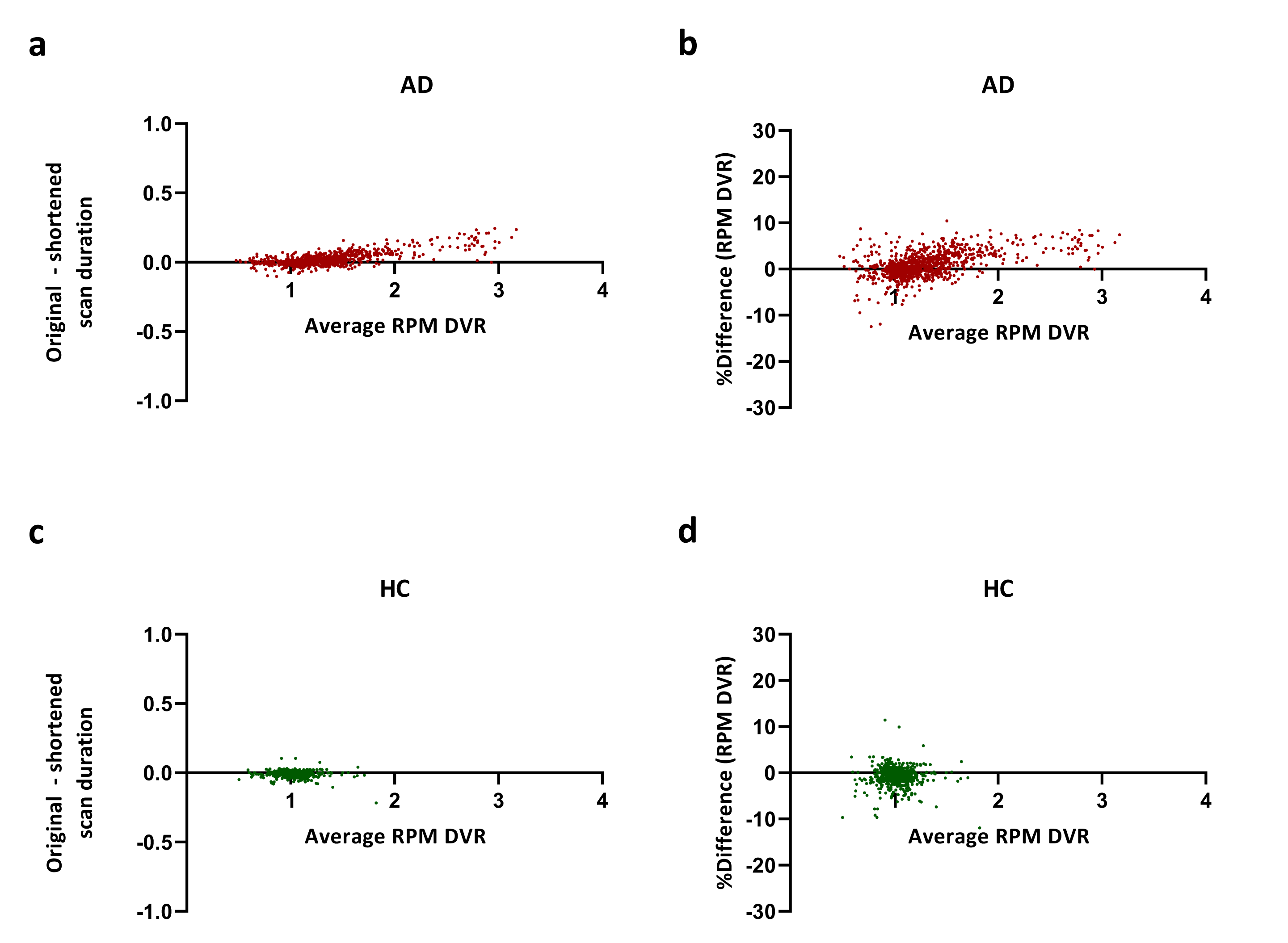

Supplement: Supplementary file 1 — (PNG 162 kb) [file 11307_2021_1581_Fig4_ESM.png]
