# Supplementary material for: Effect of Shortening the Scan Duration on Quantitative Accuracy of [18F]Flortaucipir Studies
Source: Mol Imaging Biol. 2021 Jan 26;23(4):604–13. doi: 10.1007/s11307-021-01581-5 (PMC8277654; doi:10.1007/s11307-021-01581-5)
Supplement: Supplementary file 3 — (DOCX 12 kb) [file 11307_2021_1581_MOESM2_ESM.docx]

**Supplementary Table 1.** SRTM BP_ND_ obtained using the shortened time interval (0-60/80-100) compared to plasma-input DVR-1 and SRTM BP_ND_ obtained with the original scan duration.

|  | DVR-1 (0-60/80-130) | | | | SRTM BP_ND_ (0-60/80-130) | | | |
| --- | --- | --- | --- | --- | --- | --- | --- | --- |
|  | HC | | AD | | HC | | AD | |
|  | r^2^ | Slope | r^2^ | Slope | r^2^ | Slope | r^2^ | Slope |
| SRTM BP_ND_  (0-60/80-100) | 0.96 | 0.92 | 0.91 | 1.03 | 0.99 | 1.01 | 0.99 | 0.95 |

*Note: the correspondence for the original scan duration between SRTM BP_ND_ and DVR-1 was r^2^= 0.96 slope= 0.90 for HC and, r^2^= 0.93 slope= 1.09 for AD.
