# Supplementary material for: Effect of Shortening the Scan Duration on Quantitative Accuracy of [18F]Flortaucipir Studies
Source: Mol Imaging Biol. 2021 Jan 26;23(4):604–13. doi: 10.1007/s11307-021-01581-5 (PMC8277654; doi:10.1007/s11307-021-01581-5)
Supplement: Supplementary file 4 — (DOCX 12 kb) [file 11307_2021_1581_MOESM3_ESM.docx]

**Supplementary Table 2**. RPM R_1_ obtained using shorter time intervals compared to SRTM R_1_ and RPM R_1_ obtained with the original scan duration.

|  | SRTM R_1_ (0-60/80-130) | | | | RPM R_1_ (0-60/80-130) | | | |
| --- | --- | --- | --- | --- | --- | --- | --- | --- |
|  | HC | | AD | | HC | | AD | |
|  | r^2^ | Slope | r^2^ | Slope | r^2^ | Slope | r^2^ | Slope |
| RPM R_1_  (0-60/80-120) | 0.97 | 1.00 | 0.97 | 0.99 | 1.00 | 1.00 | 1.00 | 1.00 |
| RPM R_1_  (0-60/80-110) | 0.97 | 1.00 | 0.97 | 0.99 | 1.00 | 1.00 | 1.00 | 1.00 |
| RPM R_1_  (0-60/80-100) | 0.97 | 1.00 | 0.97 | 0.99 | 1.00 | 1.00 | 1.00 | 1.00 |

Note: the correspondence for the original scan duration between RPM R_1_ and SRTM R_1_was r^2^= 0.97 slope= 1.00 for HC and, r^2^= 0.97 slope= 0.99 for AD.
