# Supplementary material for: Effect of Shortening the Scan Duration on Quantitative Accuracy of [18F]Flortaucipir Studies
Source: Mol Imaging Biol. 2021 Jan 26;23(4):604–13. doi: 10.1007/s11307-021-01581-5 (PMC8277654; doi:10.1007/s11307-021-01581-5)
Supplement: Supplementary file 5 — (DOCX 13 kb) [file 11307_2021_1581_MOESM4_ESM.docx]

**Supplementary Table 3.** Shortened time intervals interpolated using four different methods are compared with SRTM R_1_ obtained with the original scan duration.

|  |  | SRTM R_1_ (0-60/80-130) | | | |
| --- | --- | --- | --- | --- | --- |
|  |  | HC | | AD | |
|  |  | r^2^ | Slope | r^2^ | Slope |
| POP-IP 2T4k_V_B_ | SRTM R_1_  (0-50/80-100) | 0.97 | 1.01 | 0.98 | 1.01 |
|  | SRTM R_1_  (0-40/80-100) | 0.96 | 1.00 | 0.97 | 1.01 |
|  | SRTM R_1_  (0-30/80-100) | 0.95 | 1.01 | 0.96 | 1.01 |
| Cubic | SRTM R_1_  (0-50/80-100) | 0.98 | 1.01 | 0.98 | 1.01 |
|  | SRTM R_1_  (0-40/80-100) | 0.96 | 1.00 | 0.97 | 1.01 |
|  | SRTM R_1_  (0-30/80-100) | 0.95 | 1.00 | 0.96 | 1.01 |
| Linear | SRTM R_1_  (0-50/80-100) | 0.98 | 1.01 | 0.98 | 1.01 |
|  | SRTM R_1_  (0-40/80-100) | 0.97 | 1.00 | 0.97 | 1.01 |
|  | SRTM R_1_  (0-30/80-100) | 0.95 | 1.00 | 0.96 | 1.01 |
| Exponential | SRTM R_1_  (0-50/80-100) | 0.97 | 1.01 | 0.98 | 1.01 |
|  | SRTM R_1_  (0-40/80-100) | 0.96 | 1.00 | 0.98 | 1.00 |
|  | SRTM R_1_  (0-30/80-100) | 0.95 | 1.02 | 0.96 | 1.01 |
