# Supplementary material for: Effect of Shortening the Scan Duration on Quantitative Accuracy of [18F]Flortaucipir Studies
Source: Mol Imaging Biol. 2021 Jan 26;23(4):604–13. doi: 10.1007/s11307-021-01581-5 (PMC8277654; doi:10.1007/s11307-021-01581-5)
Supplement: Supplementary file 6 — (DOCX 12 kb) [file 11307_2021_1581_MOESM5_ESM.docx]

**Supplementary Table 4.** RPM R_1_ obtained with the shortened time interval (0-30/80-100) compared to SRTM R_1_  and RPM R_1_ derived from the original scan duration (0-60/80-130).

|  |  | SRTM R_1_ (0-60/80-130) | | | | RPM R_1_ (0-60/80-130) | | | |
| --- | --- | --- | --- | --- | --- | --- | --- | --- | --- |
|  |  | HC | | AD | | HC | | AD | |
|  |  | r^2^ | Slope | r^2^ | Slope | r^2^ | Slope | r^2^ | Slope |
| POP-IP  2T4k_V_B_ | RPM R_1_  (0-30/80-100) | 0.97 | 1.00 | 0.97 | 0.99 | 0.95 | 1.00 | 0.98 | 1.01 |
| Cubic | RPM R_1_  (0-30/80-100) | 0.97 | 1.01 | 0.97 | 0.99 | 0.96 | 1.00 | 0.98 | 1.01 |

*Note: the correspondence of RPM R_1_ with SRTM R_1_ for the original scan duration was r^2^ = 0.92, slope=0.95 for HC and r^2^ = 0.94, slope=0.96 for AD subjects.
